# Long-Term Remission of Primary Bone Marrow Diffuse Large B-Cell Lymphoma Treated with High-Dose Chemotherapy Rescued by *In Vivo* Rituximab-Purged Autologous Stem Cells

**DOI:** 10.1155/2012/957063

**Published:** 2012-10-16

**Authors:** Hiroshi Kazama, Masanao Teramura, Kentaro Yoshinaga, Akihiro Masuda, Toshiko Motoji

**Affiliations:** ^1^Department of Hematology, Tokyo Women's Medical University, 8-1, Kawada-cho, Shinjuku-ku, Tokyo 162-8666, Japan; ^2^Department of Surgical Pathology, Tokyo Women's Medical University, 8-1, Kawada-cho, Shinjuku-ku, Tokyo 162-8666, Japan

## Abstract

Primary bone marrow diffuse large B-cell lymphoma (DLBCL) is a rare type of extranodal lymphoma with poor prognosis. Here, we report a case of primary bone marrow DLBCL successfully treated with high-dose chemotherapy and rescued by *in vivo* rituximab-purged autologous stem cells. A 39-year-old woman visited our hospital because of anemia. Bone marrow examination revealed a large B-cell lymphoma invasion. An ^18^F-fluorodeoxyglucose positron emission tomography scan revealed disseminated bone marrow uptake without evidence of dissemination at other sites. These findings led to a diagnosis of primary bone marrow DLBCL. Our patient underwent R-CHOP (rituximab, cyclophosphamide, doxorubicin, vincristine, and prednisolone) chemotherapy and achieved complete remission. Subsequently, she received high-dose chemotherapy with an *in vivo* rituximab-purged autologous stem cell transplant. Seven years have passed since the transplantation, and she remains in remission. This suggests that transplantation of an *in vivo* rituximab-purged autograft is a promising strategy for primary bone marrow DLBCL.

## 1. Introduction

The involvement of bone marrow in diffuse large B-cell lymphoma (DLBCL) usually represents the systemic dissemination of lymphoma. However, a rare type of DLBCL involving the bone marrow alone, known as primary bone marrow DLBCL, has been reported [1–3]. The prognosis of primary bone marrow DLBCL is poor with a median survival of 14.9 months, and approximately 70% of patients die within 2 years despite receiving chemotherapy [4].

High-dose chemotherapy with autologous hematopoietic stem cell rescue is an effective treatment strategy for high-risk non-Hodgkin lymphoma [5, 6]. Furthermore, treatment with rituximab in addition to stem cell mobilization or a conditioning regimen has been reported to improve the outcome in patients with high-risk B-cell lymphoma [7]. High-dose chemotherapy rescued by *in vivo* rituximab-purged autologous stem cells is a reasonable approach for primary bone marrow DLBCL because the risk of lymphoma cells being contaminated in the autograft is high. Here, we present a case of a patient with primary bone marrow DLBCL who achieved long-term remission (at least 7 years) after high-dose chemotherapy rescued by *in vivo* rituximab-purged autologous stem cells.

The present case suggests that this strategy may enable the cure of patients with primary bone marrow DLBCL.

## 2. Case Report

A 39-year-old woman suffering from dyspnea on exertion was admitted to our hospital. On physical examination, her body temperature was 37.0°C, the conjunctiva was anemic, but neither lymphadenopathy nor hepatosplenomegaly was observed.

**Figure 1:**
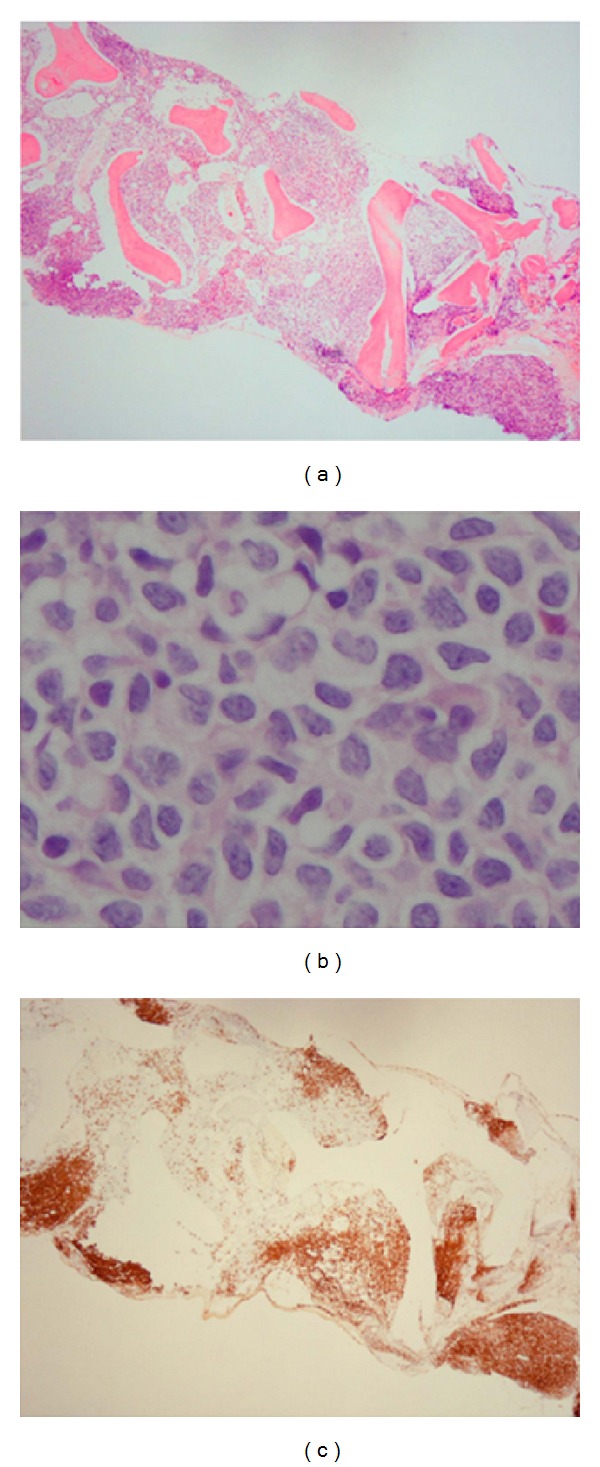
Images of the bone marrow biopsy showing the involvement of large abnormal lymphoid cells with focal clusters. (a) Hematoxylin-eosin staining, low-power field (×40); (b) hematoxylin-eosin staining, high-power field (×1,000); (c) the tumor cells were positive for CD20.

 Laboratory findings showed a decreased white blood cell count of 2,950 × 10^6^/L, a low hemoglobin level of 6.5 g/dL, an increased soluble interleukin-2 receptor level of 3,350 U/mL, and a normal serum lactate dehydrogenase level of 154 U/L. Initial bone marrow aspiration revealed a dry tap, but bone marrow biopsy showed the involvement of large abnormal lymphoid cells with focal clusters (Figures 1(a) and 1(b)). Hemophagocytosis and sinusoidal involvement of lymphoma cells were absent.

**Figure 2:**
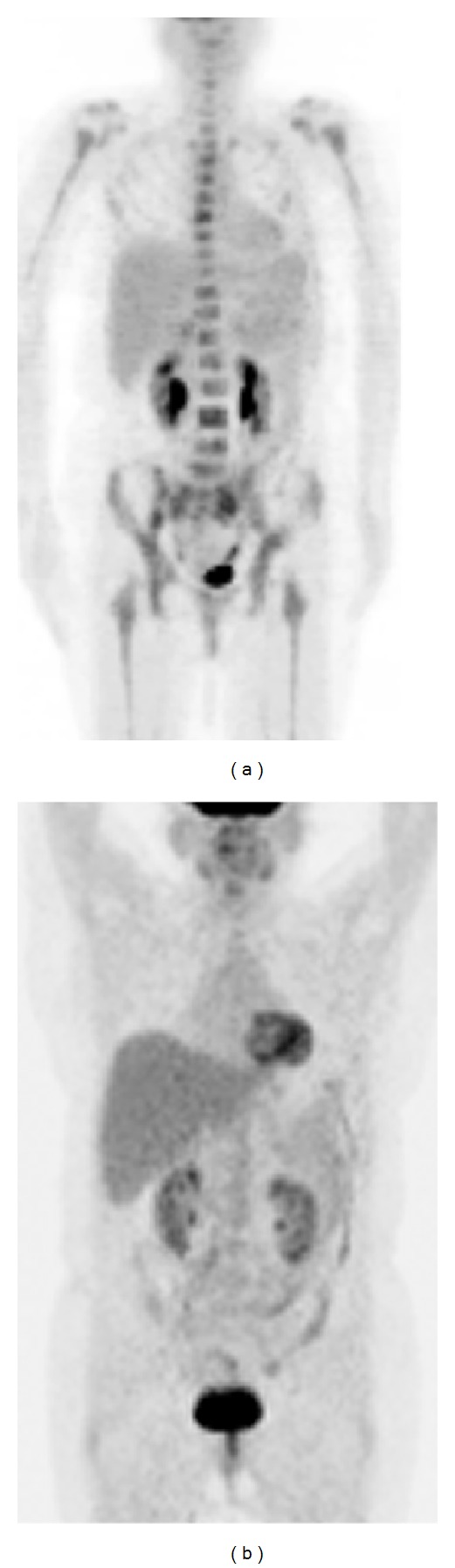
(a) An ^18^F-fluorodeoxyglucose positron emission tomography (FDG-PET) scan showing disseminated bone marrow uptake at diagnosis. (b) FDG-PET scan after autologous stem cell transplantation showing the resolution of FDG uptake.

Immunohistochemical studies indicated that the lymphoma cells were positive for CD20 (Figure 1(c)) and negative for CD3, CD5, CD34, MPO, TdT, cyclin D1, and Bcl-2. Karyotypic analysis of a second bone marrow aspiration showed a complex chromosomal abnormality: 46, XX, add (10) (q22 or q23). An ^18^F-fluorodeoxyglucose positron emission tomography scan revealed disseminated bone marrow uptake, but no abnormal uptake was observed at other sites (Figure 2(a)). These clinical findings led to a diagnosis of primary bone marrow DLBCL. The international prognostic index (IPI) score was 1.

R-CHOP (rituximab, cyclophosphamide, doxorubicin, vincristine, and prednisolone) was started, and bone marrow examination showed the disappearance of lymphoma cells after 6 courses of R-CHOP. Our patient received an additional 2 courses of R-CHOP, followed by stem cell mobilization chemotherapy consisting of 24 mg/day dexamethasone (days 1 to 10), 60 mg/m^2^ carmustine (day 2), 75 mg · m^−2^ day^−1^ etoposide (days 4 to 7), 200 mg · m^−2^ day^−1^ cytarabine (days 4 to 7), 20 mg/m^2^ melphalan (day 3), and 375 mg/m^2^ rituximab (day 1), which was started 40 days after the completion of the last course of R-CHOP. Filgrastim at a dose of 600 **μ**g was administered on day 11 and continued until the completion of leukapheresis. Leukapheresis was performed using a continuous-flow blood cell separator (Cobe Spectra, Lakewood, CO, USA), and a total of 7.3 × 10^6^ CD34^+^ cells/kg body weight were obtained.

Two weeks after stem cell collection, our patient received a conditioning regimen of 300 mg/m^2^ carmustine (day −6), 100 mg · m^−2^ day^−1^ etoposide (days −5 to −2), 200 mg · m^−2^ day^−1^ cytarabine (days −5 to −2), 140 mg/m^2^ melphalan (day −1), and 375 mg/m^2^ rituximab (day −9), followed by autologous stem cell transplantation (ASCT). All of the collected stem cells were infused, and filgrastim was administered at a dose of 450 *μ*g on days +1 to +10 and at 150 *μ*g on days +11 to +13. Engraftment of neutrophils (ANC > 1 × 10^6^/L) was performed on day +11. Seven years have passed since the transplantation and the patient remains in remission (Figure 2(b)).

## 3. Discussion

Primary bone marrow DLBCL is a rare but distinctive entity of extranodal lymphoma with a poor prognosis.

Because primary bone marrow DLBCL is rare, no standard therapy has been established. Chang et al. reported 8 cases of primary bone marrow DLBCL [3]. Four patients were treated with chemotherapy alone, of which 3 died at 8, 10, and 22 months after the initial diagnosis. In contrast, of the 4 patients treated with chemotherapy combined with rituximab (R-CHOP, 3 patients; R-COP, 1 patient), 3 have remained in remission for 3, 10, and 14 months. These results suggest that similar to other types of DLBCL, chemotherapy combined with rituximab would be a standard strategy for primary bone marrow DLBCL too. However, the long-term outcome is unknown because the followup period is too short.

Although high-dose chemotherapy rescued by autograft is known to improve the outcome of relapsed lymphoma [5], the role of upfront ASCT for the patients with DLBCL is controversial [8, 9]. Presently upfront ASCT is recommended only in selected high-risk circumstances or in the context of clinical trials [10]. Although primary bone marrow DLBCL is thought to be a type of high-risk lymphoma, the role of autologous hematopoietic stem cell transplantation is unclear because of its extremely rare incidence. To date, 3 cases of primary bone marrow DLBCL (including the present one) treated by autologous hematopoietic stem cell transplantation have been reported, all of which have remained in remission for 14 months, 12 months, and 7 years (our patient) after transplantation [3, 11].

In patients with primary bone marrow DLBCL, the high risk of lymphoma cells in the autograft being contaminated is a major problem because it may cause relapse. Therefore, an *in vivo* rituximab-purged autograft was used for transplantation in our patient. She has been alive without evidence of relapse for the past 7 years. This suggests that ASCT using an *in vivo* rituximab-purged autograft might be a promising strategy for the treatment of primary bone marrow DLBCL.

However, because this is only a case report, the role of ASCT in curing primary bone marrow DLBCL remains unclear. An accumulation of cases is required to clarify the role of ASCT with an* in vivo* rituximab-purged autograft for primary bone marrow DLBCL.
